# App-Delivered Self-Management Intervention Trial selfBACK for People With Low Back Pain: Protocol for Implementation and Process Evaluation

**DOI:** 10.2196/20308

**Published:** 2020-10-29

**Authors:** Charlotte Diana Nørregaard Rasmussen, Malene Jagd Svendsen, Karen Wood, Barbara I Nicholl, Frances S Mair, Louise Fleng Sandal, Paul Jarle Mork, Karen Søgaard, Kerstin Bach, Mette Jensen Stochkendahl

**Affiliations:** 1 National Research Centre for the Working Environment Copenhagen Denmark; 2 Department of Sports Science and Clinical Biomechanics University of Denmark Odense M Denmark; 3 Institute of Health & Wellbeing University of Glasgow Glasgow United Kingdom; 4 Department of Public Health and Nursing Faculty of Medicine and Health Sciences Norwegian University of Science and Technology Trondheim Norway; 5 Department of Clinical Research University of Southern Denmark Odense M Denmark; 6 Department of Computer Science Faculty of Information Technology and Electrical Engineering Norwegian University of Science and Technology Trondheim Norway; 7 Institute of Sports Science and Clinical Biomechanics University of Southern Denmark Odense M Denmark; 8 Nordic Institute of Chiropractic and Clinical Biomechanics Odense M Denmark

**Keywords:** randomized controlled trial, implementation, process evaluation, low back pain, digital health intervention, mHealth, decision support system, RE-AIM

## Abstract

**Background:**

Implementation and process evaluation is vital for understanding how interventions function in different settings, including if and why interventions have different effects or do not work at all.

**Objective:**

This paper presents the protocol for an implementation and process evaluation embedded in a multicenter randomized controlled trial conducted in Denmark and Norway (the selfBACK project). selfBACK is a data-driven decision support system that provides participants with weekly self-management plans for low back pain. These plans are delivered through a smartphone app and tailored to individual participants by using case-based reasoning methodology. In the trial, we compare selfBACK in addition to usual care with usual care alone.

**Methods:**

The aim of this study is to conduct a convergent mixed-methods implementation and process evaluation of the selfBACK app by following the reach, effectiveness, adoption, implementation, and maintenance framework. We will evaluate the process of implementing selfBACK and investigate how participants use the intervention in daily life. The evaluation will also cover the reach of the intervention, health care provider willingness to adopt it, and participant satisfaction with the intervention. We will gather quantitative measures by questionnaires and measures of data analytics on app use and perform a qualitative exploration of the implementation using semistructured interviews theoretically informed by normalization process theory. Data collection will be conducted between March 2019 and October 2020.

**Results:**

The trial opened for recruitment in February 2019. This mixed-methods implementation and evaluation study is embedded in the randomized controlled trial and will be collecting data from March 2019 to October 2020; dissemination of trial results is planned thereafter. The results from the process evaluation are expected 2021-2022.

**Conclusions:**

This study will provide a detailed understanding of how self-management of low back pain can be improved and how a digital health intervention can be used as an add-on to usual care to support patients to self-manage their low back pain. We will provide knowledge that can be used to explore the possibilities of extending the generic components of the selfBACK system and key drivers that could be of use in other conditions and diseases where self-management is an essential prevention or treatment strategy.

**Trial Registration:**

ClinicalTrials.gov NCT03798288; https://www.clinicaltrials.gov/ct2/show/NCT03798288

**International Registered Report Identifier (IRRID):**

DERR1-10.2196/20308

## Introduction

Self-management is often recommended as an important element of living with a chronic health condition. Self-management is defined varyingly in the literature, but key elements are “structured, multicomponent interventions that support autonomy and involve education and training with the aim of promoting adherence to self-management behaviors to achieve improved physical, psychological, and economic outcomes” [1]. Self-management involves work that many patients find challenging without support [2]. With the increased integration of digital technologies in our daily lives, digital health interventions such as smartphone apps have been suggested as promising platforms for supporting self-management [3,4], and they are increasingly being used to help people manage their chronic conditions [5]. Digital health interventions may encourage patients to engage in preventive health activities, promote communication between health care providers and patients, and improve patient adherence to treatment protocols and self-care of chronic conditions [6].

Individuals who aspire to be healthy or have more choice and control over managing their well-being are especially likely to engage with digital health interventions [7], whereas others experience barriers to engagement in digital health interventions such as poor digital literacy or negative experiences with impersonal digital health interventions [7]. Barriers and facilitators to digital health engagement are complex, and there are many interrelated factors that affect patients’ and the public’s ability to engage with digital health interventions [6,7]. Digital health interventions are more likely to achieve their full potential if they are user friendly and tailored to individual user needs [7]. However, we need to enhance our understanding of factors that hinder or promote uptake and use of digital health interventions, especially for individual conditions [6]. To gain this knowledge, systematic evaluations of implementation processes are vital for investigating how participants engage with and use digital health interventions in daily life. These evaluations consist of multiple components, which together can help distinguish between interventions that are inherently faulty (failure of intervention concept or theory) and those that are poorly delivered (implementation failure) [8]. Information regarding implementation (eg, delivery and receipt of the intervention) is an important aspect in a process evaluation [9] and can provide valuable insights regarding likely future implementation ability of the digital health intervention in real-world settings.

Low back pain is a very common condition and the most significant contributor to years lived with disability and disability-adjusted life years globally [10,11]. In addition to the individual health consequences, low back pain poses an enormous economic burden on the European economy [10,11], which is only expected to increase as the European population ages. In most people a specific cause of low back pain will not be identified; thus, most low back pain is termed nonspecific, and for many, the condition becomes recurrent or long-lasting [12]. Evidence-based guidelines for treatment of nonspecific low back pain consistently endorse self-management as a central part of low back pain care [13-16]. Self-management in chronic conditions may be supported by digital health interventions, but in relation to low back pain the current evidence is weak [17]. Published studies have been heterogeneous with poor descriptions of interventions and limited theoretical underpinnings suggesting the need for further research in this sphere [17,18]. In the burgeoning field of digital health interventions, it therefore seems relevant to develop high-quality digital self-management interventions for people with low back pain [17-19] and evaluate the process of implementing the interventions to gain insights into why digital self-management interventions work or do not work, and how they can be optimized to increase likelihood of success [7,20].

The selfBACK study aims to create and implement a smartphone app with high-quality, evidence-based content that provides self-management support to people with low back pain [21,22]. This paper describes the protocol for an implementation and process evaluation study embedded in a multicenter, randomized controlled trial (RCT) of the selfBACK app compared with usual care. The focus of this paper is the planned evaluation of the implementation processes. A detailed protocol concerning the design of the RCT and a feasibility study have been reported elsewhere [23,24]. Results of the pilot study will be reported separately.

## Methods

### Aims of This Study

We will conduct a convergent mixed-methods implementation and process evaluation of the selfBACK app by following the reach, effectiveness, adoption, implementation, and maintenance (RE-AIM) framework [25].

The specific aims of the study according to the RE-AIM framework are as follows:

- Describe the proportion and characteristics of participants and nonparticipants in selfBACK and the recruitment pathways
- Evaluate self-perceived effect and user acceptability and satisfaction using quantitative measures combined with interview-based explorations of both participant and health care provider appraisals of selfBACK
- Describe health care provider recruitment strategies of potential participants and identify factors affecting uptake of health care provider adoption of selfBACK
- Explore the implementation of selfBACK and how participants embed and integrate use of the app in daily routines, and compare participants with different levels of engagement and use
- Explore participants’ intended future use and sustained engagement with the selfBACK app

### selfBACK Overview

The selfBACK project is a 5-year project (2016-2020) funded by the European Union Horizon 2020 Research and Innovation Programme. In brief, the selfBACK intervention is a data-driven decision support system that provides participants with tailored, weekly updated self-management plans delivered through a smartphone app. The app content builds on clinical guidelines for treatment of low back pain and has three main components: (1) physical activity advice and step counting using input from an activity-detecting wristband, (2) education based on a cognitive behavioral approach, and (3) instructions on physical strength and flexibility exercises. The content of the app used is described in detail in the protocol paper [21-23]. Tailoring of the self-management plans is achieved by using the case-based reasoning methodology, which is an artificial intelligence method. In selfBACK, the case-based reasoning system takes data about the current case (participant) and compares it with data from previous successful ones to find similar cases (participants) that are used to tailor the self-management plan for the current case (participant). The intervention will be tested in a multicenter RCT with two parallel arms conducted in Denmark and Norway. The trial period is 9 months with primary outcome (pain-related disability) assessed at 3 months. The control arm will receive usual care (ie, follow any diagnostic or treatment-related pathway as instructed by their health care provider). The intervention arm will use the selfBACK app in addition to usual care. The trial will include 350 participants allocated 1:1 to the usual care arm and intervention arm (selfBACK as an add-on to usual care) [21-23]. Eligible participants for the RCT are individuals who seek care from a primary health care practice or an outpatient hospital facility for nonspecific low back pain.

In both Norway and Denmark, participants are recruited from general practice, physiotherapy, and chiropractic clinics. Additionally, in Denmark participants are also recruited from the Spine Centre in the region of Southern Denmark. The Spine Centre is a specialized outpatient hospital facility that reviews patients with back pain referred from primary care. The Spine Centre provides diagnostic assessment of patients and prescribes treatment plans according to national treatment guidelines. Patients seen at the Spine Centre without serious pathologies may be referred to the selfBACK study.

The recruitment period for the RCT started in March 2019. In each country, collaborations with local clinics and health care providers were established to facilitate recruitment. Health care providers refer potentially eligible participants based on a short description of eligibility. Final eligibility is assessed by the research team during a screening phone call.

### Ethical Approval and Consent to Participate

National ethics approvals have been granted for both the Danish (Regional Scientific Ethical Committee for Southern Denmark, S-20182000-24) and Norwegian (Regional Committee for Medical and Health Research Ethics, 2017/923-6) sites of the RCT, including the process evaluation. Regarding collecting, managing, and storage of data, approval was granted from the Danish Data Protection Agency through application to the University of Southern Denmark’s legal office (201-57-0008) and from the Norwegian National Data Protection Authority and the Centre for Research Data through ethics approval. The trial was registered with ClinicalTrials.gov [NCT03798288].

All participants are asked for informed consent, assigned after the principles of the Helsinki Declaration II. All data will be treated confidentially and stored in pseudoanonymized form. The quantitative data will be stored on a server in Norway, while the interview data will be stored in Denmark. Both servers are secure and firewall-protected, and backups are performed daily. In both countries, data handling and storage is consistent with the European regulations on data protection.

### Guiding Theoretical Frameworks

This implementation and process evaluation study integrates three published frameworks to guide the design of the intervention and formative evaluation: (1) intervention mapping [26], used to conceive and develop the intervention; (2) the RE-AIM framework [25,27], used to guide the overall evaluation of the study and assess implementation success of the selfBACK app [25,27]; and (3) normalization process theory [28], used to guide the evaluation of barriers and facilitators that may affect implementation, providing a more detailed understanding of how and why the trial achieves the observed results.

### Intervention Mapping

Complex innovations such as digital health behavior change interventions can be conceived and developed using this comprehensive framework [26]. Intervention mapping has been described as “providing a systematic and stepwise approach to planning interventions” [29]. Intervention mapping enables identification of behavioral and environmental determinants likely to influence engagement with and operationalization of recommended self-management behaviors, thus enhancing the potential for intervention success. The development of the intervention using intervention mapping is described in brief elsewhere [23].

### RE-AIM Framework

Our overall evaluation is guided by the RE-AIM framework and investigates all five elements of the framework: reach, effectiveness, adoption, implementation, and maintenance [25,27]. The RE-AIM framework is an evaluation framework that aims to determine the success of an intervention implementation within a given context [25,30]. It has been extensively used in RCTs to evaluate the external validity and sustainability of effective practices [31] including digital health interventions in diabetes [32] and mental disorders [33].

### Normalization Process Theory

Normalization process theory [28] is an implementation theory [30] that provides a framework for the collection and analysis of data and a coherent set of explanations of implementation processes [34]. Using this theoretical framework will enable us to identify, characterize, and explain mechanisms that shape the implementation process of selfBACK, which will in turn influence outcomes [34]. Normalization process theory has increasingly been used as a framework in prospective evaluations of health care innovations or interventions, particularly digital health interventions [35], as part of service deployments or clinical trials. Normalization process theory provides a conceptual framework that enables increased understanding of the factors that influence how new technologies or therapies become implemented, embedded, and integrated, or not, into routine use or everyday life [34]. It has also been used extensively to understand self-management practices of patients [36].

### Data Collection and Analysis

This mixed-methods implementation and evaluation study is embedded in the RCT and will be collecting data from March 2019 to October 2020. We will gather self-reported data by questionnaires (including characteristics of the participants such as age, sex, and working status), retrieve data analytics on app use, and perform a theoretically informed qualitative exploration involving semistructured interviews with participants and health care providers.

### Interview Participants

Intervention arm participants in both Denmark and Norway will be selected for interview based on a simple measure of adherence to the intervention: number of weekly self-management plans generated during the first 3 months of the intervention period. During this period and dependent on participants’ adherence, between 1 and 14 plans may be generated. Cut points for 3 adherence level groups will be based on pilot data of participant app use and defined as 1 to 7 plans (low or nonuse), 8 to 12 plans (moderate use), and 13 to 14 plans (high use). Approximately 6 to 8 participants from each of the 3 groups in the intervention arm will be interviewed [37]—up to 24 interviews or until data saturation is reached.

In addition, 6 to 8 participants in the usual care group will be interviewed. Even though the intervention group allocation in the RCT will be 1:1, the number of participants interviewed from the usual care arm will be lower since interviews will only pertain to general experiences of low back pain self-management and perceived effect of participation in the trial. The majority of the interviews will be conducted in Denmark as this is the primary country of patient recruitment.

The number of health care providers to be interviewed will be determined based on the number of recruitment sites needed for the RCT. To secure maximum variation, we will interview health care providers from all participating professions and aim to identify health care providers with varying success in terms of recruitment to the RCT. The estimated sample size is approximately 10 health care providers. Interviews will be undertaken either in person or via telephone and will be audiotaped with participant or health care provider consent and transcribed verbatim to provide data for the qualitative analyses.

### RE-AIM Components

Textbox 1 provides a detailed description of the activities related to each of the five elements in the RE-AIM framework.

**Description of quantitative and qualitative data collection strategies.**

Reach

- Quantitative:
  Participant recruitment flow: number of referred, screened, enrolled, and randomized; reason for nonparticipation or exclusion
  Participant characteristics: sociodemographic data; fear avoidance; self-efficacy; illness perception
  Recruitment strategy: description of recruitment pathways into selfBACK
- Qualitative:
  Semistructured interviews, intervention arm: experience of enrollment in selfBACK
  Semistructured interviews, usual care arm: experience of enrollment in selfBACK

Effectiveness

- Quantitative:
  User satisfaction, intervention arm: Virtual Care Climate Questionnaire plus overall rating items
- Qualitative:
  Semistructured interviews, intervention arm: motivation for participating; perception of self-management; change in self-management behavior; effect of participating; satisfaction and appraisal of selfBACK app
  Semistructured interviews, usual care arm: motivation for participating; perception of self-management; change in self-management behavior; effect of participating (if any)
  Semistructured interviews or focus groups, health care providers: perception and appraisal of selfBACK

Adoption

- Quantitative:
  Recruiter flow: numbers of invited and accepting health care providers; number of patients informed about the selfBACK study per health care provider or clinic
  Characteristics of health care providers: type; number of health care providers per clinic
  Recruitment strategy: description of how clinics or health care providers were recruited to selfBACK
- Qualitative:
  Semistructured or focus groups interviews, health care providers: role in patient engagement; success of engagement; barriers and facilitators for informing patients about the study

Implementation

- Quantitative:
  App use data, 0 to 3 months: frequency of use; number of app visits; time spent using the app; number of days with visits; number of plans generated; goal achievement scores
  Participant characteristics: sociodemographic data; fear avoidance; self-efficacy; illness perception
- Qualitative:
  Semistructured interviews, intervention arm: attitude toward self-management of low back pain and the selfBACK app; barriers and facilitators for engagement; experience of using selfBACK app; challenges of engaging with selfBACK app and what helped; embedment in daily routine

Maintenance

- Quantitative:
  App use data, 4 to 9 months: frequency of use; number of app visits; time spent using the app; number of days with visits; number of plans generated; goal achievement scores
- Qualitative:
  Semistructured interviews, intervention group: perspectives on intended sustained engagement with selfBACK app

### Reach

The first dimension of interest in the RE-AIM framework is the reach of the intervention, which refers to the proportion of the target population participating in the intervention [25]. This will provide valuable information about interest in the intervention, eligibility rates among those interested in using the selfBACK system, and details on why some interested respondents are deemed ineligible.

The selfBACK RCT follows the recommendations outlined in the Consolidated Standards on Reporting Trials (CONSORT) guidelines [38]: number of invitations for trial participation and acceptance rates and basic sociodemographic variables for all patients screened for eligibility. Recruitment pathways and flow of recruitment (eg, invitations and acceptance rates) will be examined to see how many people fail the eligibility screening and how many proceed to the trial. During the interviews, we will ask participants about their experience in the enrollment process.

### Effectiveness

The second dimension of the RE-AIM framework is effectiveness, which refers to the impact, including potential negative effects, of the intervention on important outcomes [25]. We evaluate effectiveness from the participant perspective using measures of self-perceived effect and user acceptability and satisfaction. This will be investigated both quantitatively, through self-reported patient questionnaires, and qualitatively, through questionnaire data and semistructured interviews. The primary outcome of the RCT, pain-related disability measured by the Roland Morris Disability Questionnaire [39,40] at 3 months, and a range of secondary outcomes [23] will be reported separately from the implementation evaluation.

Quantitatively, we will collect data on effectiveness approximately 4 months after baseline using a web-based questionnaire including the 15-item version of the Virtual Care Climate Questionnaire (VCCQ), which evaluates how participants perceive the effectiveness of the autonomy-supportive communication for health behavior change offered in a virtual care setting [41].

The VCCQ has a 7-point response scale with totally disagree and totally agree as end points. Further, 3 items on overall rating of the selfBACK app, ease of use of the app, and recommendation to others will be used, rated using a 5-point rating system in the same way commercial apps often do. The VCCQ and the rating items will be sent only to the intervention group. To complement participant perspectives on effectiveness, health care provider perceptions and appraisals of the selfBACK system in terms of its value to practice will also be investigated through interviews. Semistructured interviews with participants from the intervention arm will be performed to investigate their motivation for participating in the trial, perceptions of self-management, views on acceptability and satisfaction with the app, and appraisal of the effects, if any, of use of the selfBACK system. Usual care arm interviews will likewise elucidate participant motivation for participating in the trial, their perception of self-management, and any effect participation has induced.

### Adoption

Adoption—the third dimension of the RE-AIM framework—refers the willingness of health care providers to inform patients about the selfBACK intervention along with perceived barriers and facilitators to participation and recruitment [25]. Adoption will be assessed by investigating which potential clinical sites adopt the intervention (ie, agree to participate in the trial and inform patients about the study). Semistructured interviews with purposefully sampled health care providers will be undertaken to explore how the health care providers engaged patients (eg, what informal criteria they used for selecting which participants to inform about the study and what barriers and facilitators for engaging patients they experienced). Further, strategies employed by the researchers to recruit health care providers to the selfBACK trial and sustain health care provider engagement of patients into the trial will be described narratively.

### Implementation

Implementation is the fourth dimension of the RE-AIM framework and describes to what extent the intervention is implemented as intended [25]. This is usually described as examining how effectively and consistently an intervention is delivered in a specific context (eg, primary or secondary care) by staff. As the selfBACK intervention is not being delivered by staff but instead through a self-management app, we will focus on how the selfBACK app becomes embedded and integrated into the daily routines of participants with low back pain.

When assessing implementation, we will focus on the first 4 months of the intervention. Semistructured interviews with participants in the intervention group will be undertaken with participants with different levels of use of the app (ie, high, moderate, low, and nonuse). The interviews will focus on how and why the participants embed and integrate the selfBACK app in daily life or do not (eg, their attitude toward self-management of low back pain, barriers and facilitators for engagement, and experience with using the selfBACK app). Interviews will be conducted approximately 4 months after inclusion (ie, after participants have completed the follow-up questionnaires that feature the primary outcome and VCCQ). The interviewer will be blinded to the outcome measures.

Implementation will also be assessed quantitatively by investigating app use analytics data (eg, frequency of use and goal achievement scores) throughout the first 3 months after inclusion. Information on individual participant app use will be drawn from the backend system serving selfBACK. The app use data will also be used to compare the groups of participants with different adherence levels on characteristics such as sociodemographic data, fear avoidance, self-efficacy, and illness perception.

### Maintenance

Maintenance is the fifth dimension of the RE-AIM framework and refers to the extent to which engagement with the intervention is sustained at the individual and system level over time [25]. Due to the nature of the selfBACK intervention, our focus will be on the concept of maintenance at the individual level: use of the app beyond 3 months and up to 9 months (full trial period). We will examine this with app analytics for the trial period 4 to 9 months after baseline and explore participants’ intended future use beyond the 3 months through semistructured interviews with intervention arm participants.

### Normalization Process Theory Components

**Figure 1:**
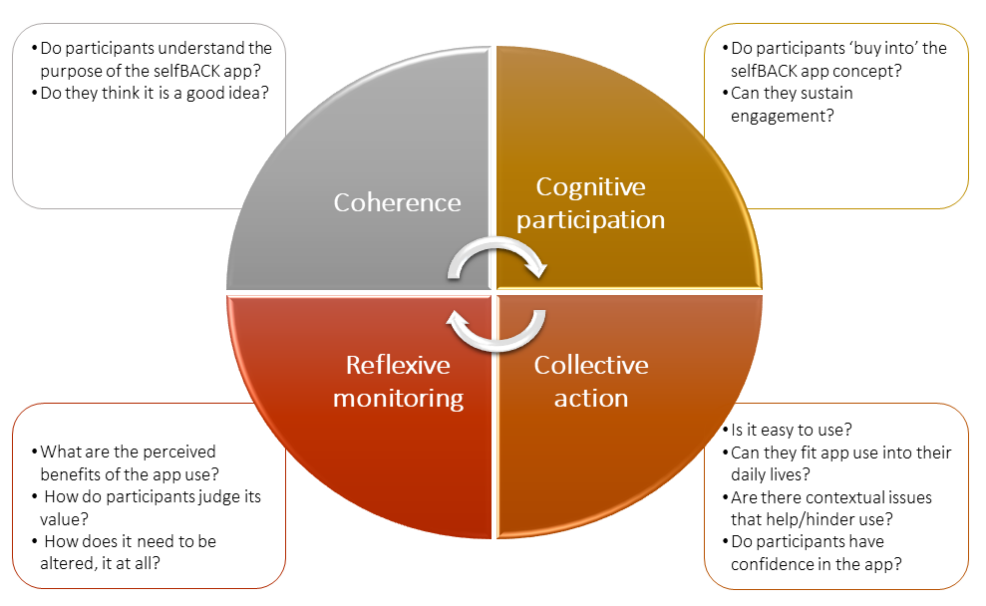
Four constructs of normalization process theory and associated questions from the process evaluation.

In Figure 1, we have outlined the four main constructs of normalization process theory that will guide the collection and analysis of interview data in the selfBACK RCT.

Coherence refers to the sense-making work individuals undertake that influences whether they are willing to embed a new practice in their lives and will influence initial adoption of the intervention by health care providers and participants using the app. Specifically, we will explore whether participants understand the purpose of the selfBACK app and if they like the idea.

Cognitive participation is the work individuals undertake to engage with the new practice and will influence implementation. Participants will be prompted to consider their level of buy-in of the selfBACK app concept and potential for sustained engagement.

Collective action refers to the work individuals do to enact a new practice, which will be related to implementation and maintenance. We will elucidate participant confidence in using the app and integrating use in their daily lives. Further, any contextual issues that help or hinder the participant will be highlighted.

Reflexive monitoring is the appraisal work individuals undertake to determine whether the new practice is worth sustaining or how it must be reconfigured to fit their needs and will relate particularly to maintaining their use of the intervention. Participants will be asked about the perceived benefits, if any, of using the app, its value to them, and the need for any modifications.

### Analysis

#### Quantitative Data Analysis

Simple descriptive statistics will be used to do comparisons and test for differences on questionnaire and app use data. Statistical analyses will be performed in the most recent version of SPSS Statistics (IBM Corporation).

#### Qualitative Data Analysis

Qualitative data will be analyzed using a framework approach underpinned by normalization process theory. In terms of usual care participants, normalization process theory will serve to help understand experiences of low back pain and facilitators and barriers to low back pain self-management in general. The analyses will follow the five stages of framework analysis described by Ritchie [42]: familiarization, identifying a thematic framework, indexing, charting, and mapping and interpretation. The distribution of codes will be recorded and, importantly, any data that fall outside of the coding frame will be identified and examined to determine if important concepts or ideas are being missed by using the chosen theoretical framework. Transcripts will be analyzed and coded in their original language. To ensure consensus on themes and coding, coding meetings will be arranged to discuss coding among key researchers. At the meetings, the coding framework and a proportion of the data will be double coded to ensure data analysis is robust and coders are open to identifying themes that fall outside the normalization process theory framework. In this way, coding will be iterative and responsive to the data and inappropriate shoe-horning of the qualitative data collected will be avoided.

## Results

Recruitment to the trial started in early 2019 and ran until the end of 2019. Data collection started in March 2019 and is expected to be complete by October 2020; dissemination of trial results is planned thereafter. The results from the process evaluation are expected in 2021-2022.

## Discussion

This mixed-methods implementation and process evaluation embedded in an RCT will assess the factors that influence uptake and use of a digital health intervention for self-management of low back pain as an addition to usual care. In addition to identification of factors that influence the effect of the selfBACK intervention, we will explore participant acceptance and patterns of use of the selfBACK app in the intervention arm and attempt to understand current self-management strategies for those in the usual care arm. In preparation for this study, we aimed to ensure high usability of selfBACK by involving people with low back pain and health care providers during the development phase of the project. In our evaluation, we will extend on the development process and assess health care provider experiences with the selfBACK system and their views on future implementability. Further, we have undertaken an intervention mapping process, developed logic models of change (described elsewhere) [43] and described the theoretical underpinnings for the selfBACK system. However, our process evaluation will enable an in-depth examination of factors identified as key by the normalization process theory, particularly factors that influence uptake of a digital health intervention directed at self-management and factors related to the integration of the selfBACK intervention into daily life.

This study will provide a detailed understanding of how self-management of low back pain can be improved and how a digital health intervention can be used as an add-on to usual care to support patients to self-manage their low back pain. We will provide knowledge that can be used to explore the possibilities of extending the generic components of the selfBACK system and key drivers that could be of use to other conditions and diseases where self-management is an essential prevention or treatment strategy such as diabetes, osteoarthritis, rheumatoid arthritis, cardiovascular disease, or chronic obstructive pulmonary disorders. It will also provide valuable insights into health care provider views and likely improve future implementability of the selfBACK system at scale to support self-management of low back pain. Last, this thorough implementation and process evaluation integrated in the RCT will enhance the credibility of the findings from our trial and provide important input for improving the selfBACK system in order to enhance its future impact.

## Abbreviations

CONSORT: Consolidated Standards on Reporting Trials
RCT: randomized controlled trial
RE-AIM: reach, effectiveness, adoption, implementation, and maintenance
VCCQ: Virtual Care Climate Questionnaire
